# Supplementary material for: RNP components condense into repressive RNP granules in the aging brain
Source: Nat Commun. 2022 May 19;13:2782. doi: 10.1038/s41467-022-30066-4 (PMC9120078; doi:10.1038/s41467-022-30066-4)
Supplement: Supplementary file 1 — Supplementary Information [file 41467_2022_30066_MOESM1_ESM.pdf]

Supplementary Information

RNP components condense into repressive RNP granules in the aging brain

Pushpalatha et al.

Supplementary Figures 1-11

Supplementary Data 1-2

Pushpalatha et al., Supplementary Fig. 1

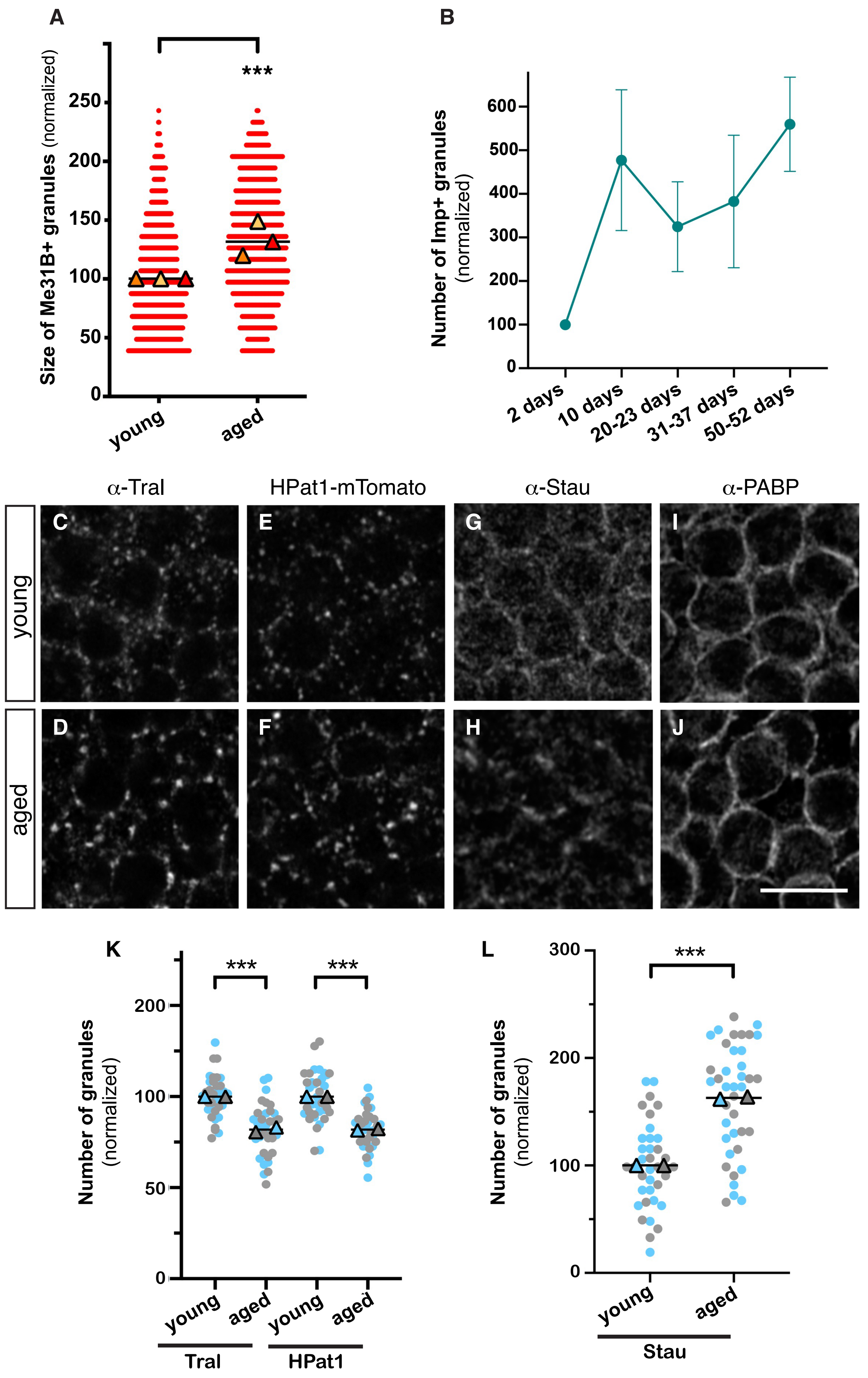

Supplementary Figure 1. Clustering of RNP granule components

(A) Sizes of Me31B-positive granules in 1-2 day-old (young) and 37-38 day-old (aged) control brains. Values were normalized to the young condition. Three replicates were performed and the mean value of each replicate is indicated as a symbol (triangle). The distribution of granule sizes is shown for one replicate only. ***, $P<0.001$ (unpaired, two-sided t-test). (B) Mean number of Imp-containing granules upon gradual aging (per surface area, normalized to 1-2 days). Three replicates were performed per condition. At least 15 samples were analyzed per condition. Error bars represent s.e.m. (C-J) Cell bodies of MB γ neurons imaged from 1-2 day- (young; C,E,G,I), or 37-38 day- (aged; D,F,H,J) old brains. Brains were stained with anti-Tral (C,D), anti-Staufen (G,H) or anti-PABP (I,J) antibodies. Signal produced by a HPat1-mTomato knock-in line is shown in E,F. Scale bar: 5 μm. (K-L) Numbers of granules containing Tral (K; left), HPat1 (K; right) or Staufen (L). Values were normalized to the young condition. Note that age-dependent condensation of Tral and HPat1 results in a decrease number of detected granules (similarly to what is observed with Me31B). Age-dependent condensation of Staufen, in contrast, leads to an increase in the number of granules that can be detected over the diffuse cytoplasmic signal (similarly to what is observed with Imp). Two replicates were performed and the mean value of each replicate is indicated as a symbol (triangle). At least 18 fields were imaged per condition. ***, $P<0.001$ (unpaired, two-sided t-tests). Source data are provided as a Source Data file.

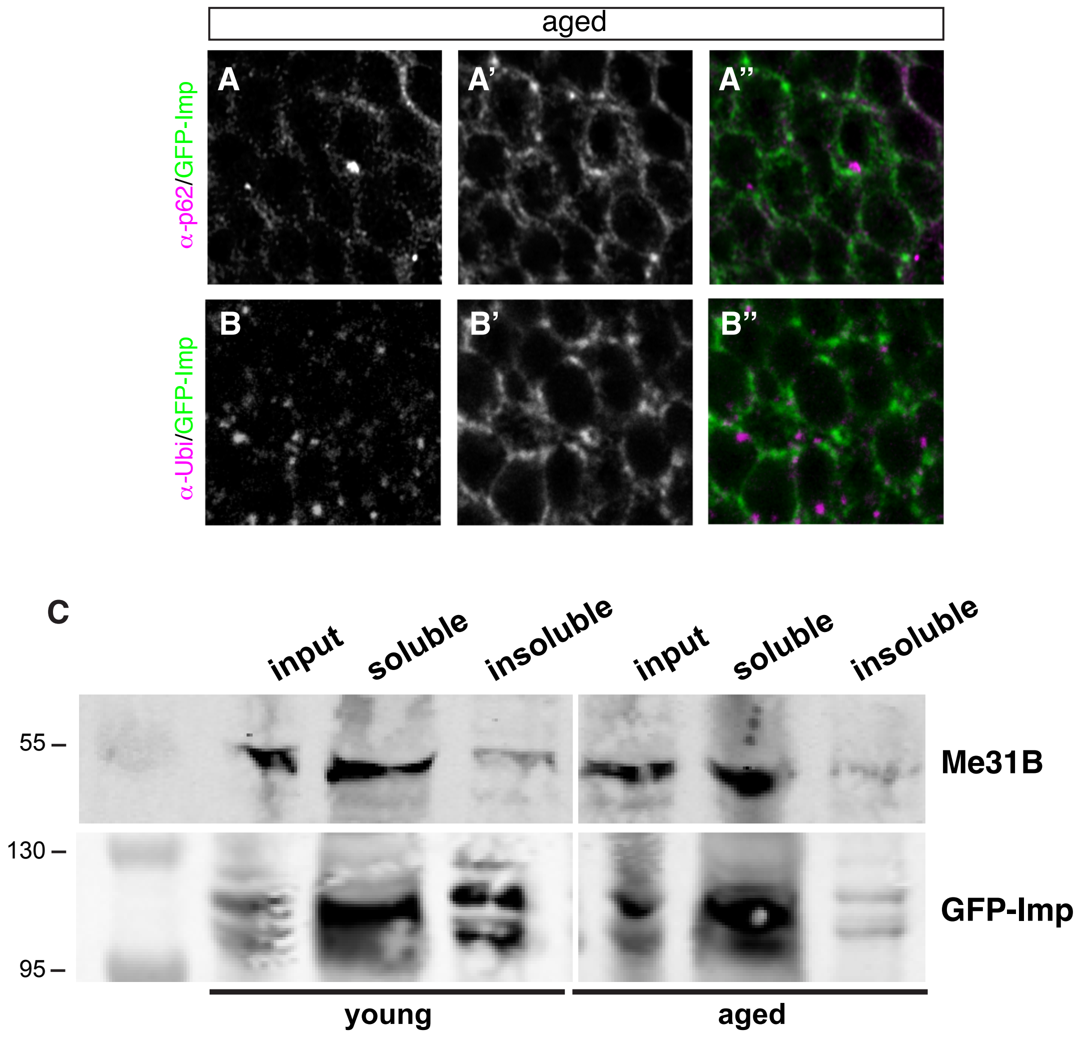
aged
A
A'
A''
B
B'
B''
α-p62/GFP-Imp
α-Ubi/GFP-Imp
C
input
soluble
insoluble
input
soluble
insoluble
55
130
95
Me31B
GFP-Imp
young
aged

Supplementary Figure 2. Imp and Me31B do not assemble into insoluble protein aggregates in aged brains.

(A,B) Cell bodies of MB γ neurons imaged from 37-38 day-old (aged) brains. GFP-Imp-expressing brains were stained with anti-p62 (A, magenta in A'') or anti-Ubiquitin (B, magenta in B'') antibodies. GFP-Imp distribution is shown in white in A',B' and in green in A",B". Two independent replicates were performed. Scale bar: 5 μm. (C) Western-Blot performed with soluble and insoluble fractions recovered from young and aged GFP-Imp-expressing brain lysates. No increase in the amount of GFP-Imp and Me31B proteins found in the insoluble fraction was observed upon aging. Two independent replicates were performed. Source data are provided as a Source Data file.

Pushpalatha et al., Supplementary Fig. 3

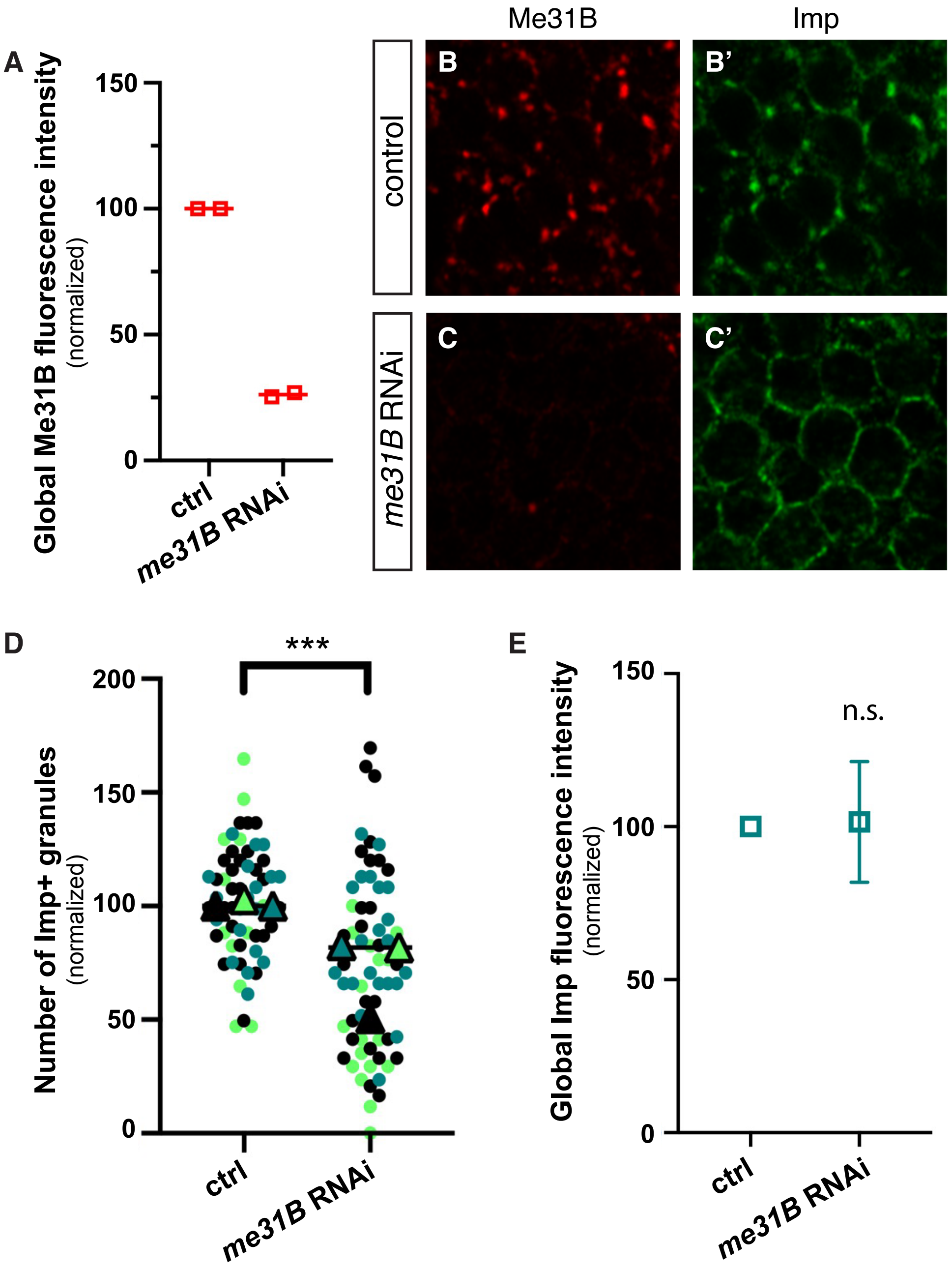

Supplementary Figure 3. *me31B* is required for the recruitment of Imp to granules.

(A) Relative total amount of Me31B in MB γ neurons from control and *me31B* RNAi brains. Data were normalized to the control values. Each data point represents the mean value obtained from one replicate. Two independent replicates were performed. (B,C) Cell bodies of 35-38 day-old GFP-Imp-expressing MB γ neurons stained with anti-Me31B (B,C) antibodies. GFP-Imp signals are shown in B',C'. Cell bodies shown in C,C' were subjected to *me31B* RNAi. Scale bar: 5 μm. (D) Number of Imp+ granules (per surface area, normalized) in control and *me31B* RNAi aged brains. Three replicates were performed and the mean value of each replicate is indicated as a symbol (triangle). Data points were color-coded based on the experimental replicate they belong to. At least 12 fields were imaged per condition. ***, $P<0.001$ (unpaired, two-sided t-test). (E) Total amount of Imp in MB γ neurons from control and *me31B* RNAi brains. Data were normalized to the control values. Each data point represents the mean value obtained from four independent replicates. Error bars represent s.e.m. n.s. stands for not significant (unpaired, two-sided Mann-Whitney test on replicate means). Complete genotype: UAS-*me31B*-RNAi/tub-Gal80ts;;OK107-Gal4/+. Source data are provided as a Source Data file.

Pushpalatha et al., Supplementary Fig. 4

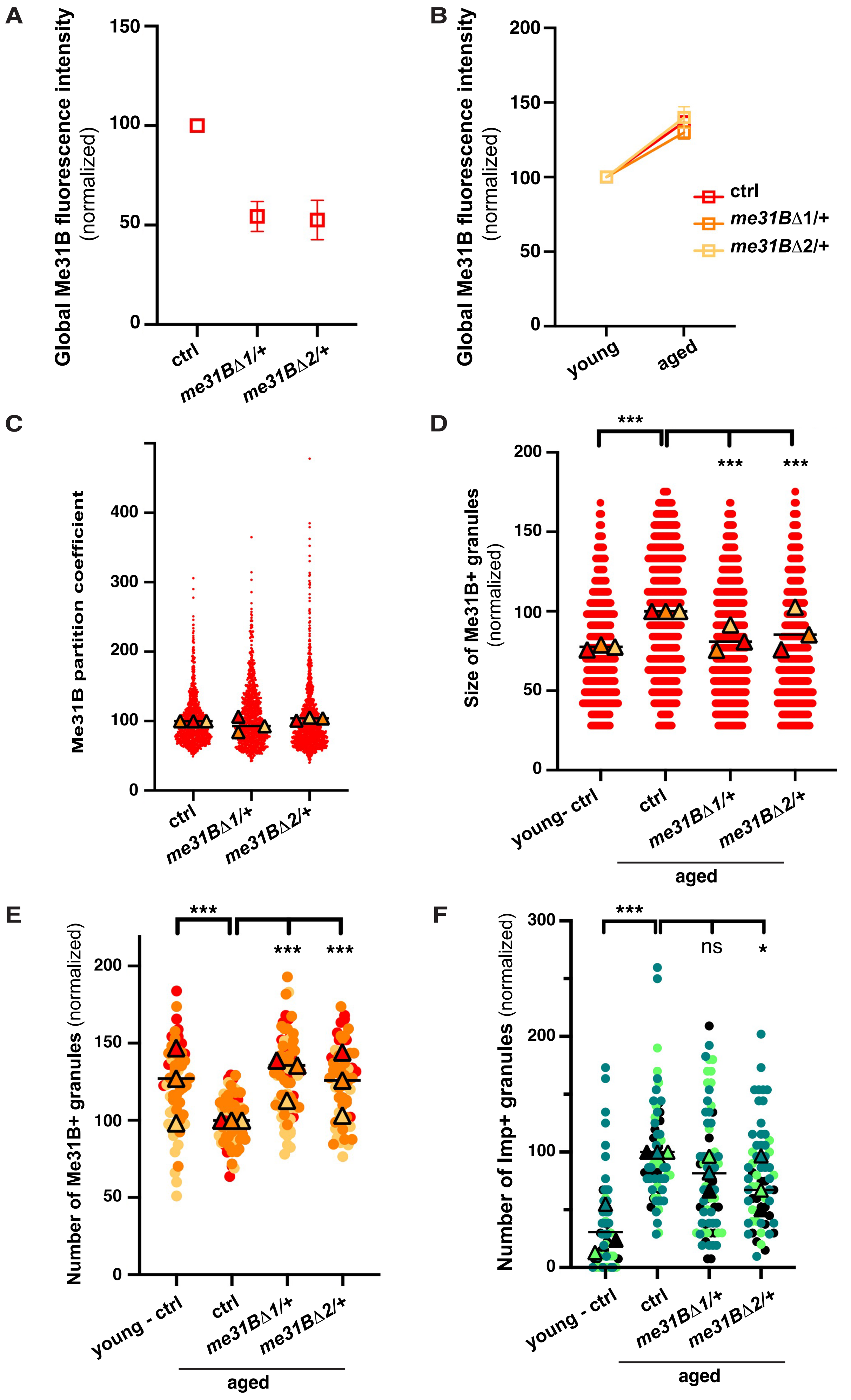

Supplementary Figure 4. Me31B levels and granule properties in *me31BΔ*/+ individuals.

(A) Me31B-GFP levels measured from 35-38 day-old MB γ neurons. Values were normalized to the control condition. (B) Me31B-GFP levels measured from 1-2 day- (young) and 35-38 day- (aged) old MB γ neurons. Values were normalized to the young condition. In A,B, data points represent the mean values obtained from three independent replicates and error bars s.e.m. At least 12 fields were imaged per replicate and per condition. (C) Partition coefficient of Me31B-GFP in 37-38 day- (aged) old brains of controls and heterozygous (*me31BΔ1*/+ or *me31BΔ2*/+) brains. Partition coefficients were estimated by dividing the maximal intensity of Me31B signal in individual RNP granules to the intensity of the cytoplasmic diffuse pool (see Methods), and calculated for each granule detected in the imaged fields. Three replicates were performed and the mean value of each is indicated as a triangle. The distribution of individual granule partition coefficients is shown for one replicate only. (D,E) Distribution of sizes (D) and number (E) of Me31B-containing granules in control (ctrl) and heterozygous (*me31BΔ1*/+ or *me31BΔ2*/+) brains from 1-2 day- (young) or 37-40 day- (aged) old flies. Values were normalized to the aged control condition. In C-E, three replicates were performed and the mean value of each is indicated as a triangle. In C-D, the distribution of granule sizes is shown for one replicate only. In E, data points were color-coded based on the replicate they belong. At least 12 fields were imaged per replicate. ***, $P<0.001$ (one-way ANOVA test with Sidak's multiple comparison tests, performed on individual data points). (F) Numbers of Imp-containing granules (per surface area; normalized to aged controls). Three replicates were performed and the mean value of each is indicated as a triangle. Data points were color-coded based on the replicate they belong to. At least 10 fields were imaged per condition. *, $P<0.05$; ***, $P<0.001$ (one-way ANOVA test with Sidak's multiple comparison tests). n.s. stands for not significant. $P=0.03$ for *me31BΔ2*/+ and 0.08 for *me31BΔ1*/+. Source data are provided as a Source Data file.

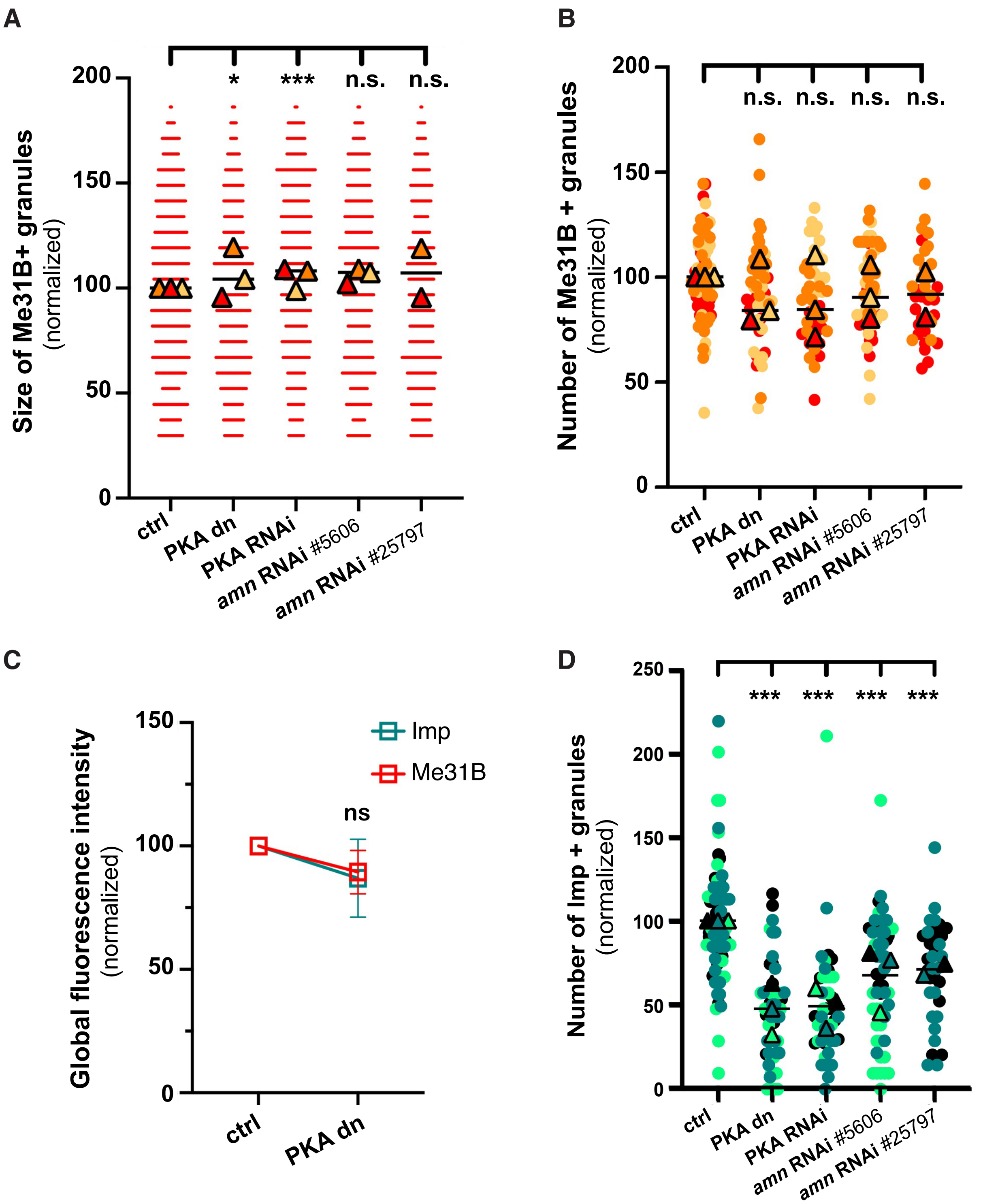
A
Size of Me31B+ granules
(normalized)
0
50
100
150
200
*
***
n.s.
n.s.
ctrl
PKA dn
PKA RNAi
amn RNAi #5606
amn RNAi #25797
B
Number of Me31B + granules
(normalized)
0
50
100
150
200
n.s.
n.s.
n.s.
n.s.
ctrl
PKA dn
PKA RNAi
amn RNAi #5606
amn RNAi #25797
C
Global fluorescence intensity
(normalized)
0
50
100
150
Imp
Me31B
ns
ctrl
PKA dn
D
Number of Imp + granules
(normalized)
0
50
100
150
200
250
***
***
***
***
ctrl
PKA dn
PKA RNAi
amn RNAi #5606
amn RNAi #25797

Supplementary Figure 5. PKA inactivation does not significantly affect Me31B condensation. Distribution of sizes (A) and number (B) of Me31B-containing granules in 30 day-old (aged) control brains and brains with reduced PKA activity. Values were normalized to the control condition. Three replicates were performed and the mean value of each replicate is indicated as a symbol (triangle). In A, the distribution of granule sizes is shown for one replicate only. In B, data points were color-coded based on the experimental replicate they belong. *, $P<0.05$; ***, $P<0.001$ (one-way ANOVA test with Sidak's multiple comparison tests, performed on individual data points). n.s. stands for not significant. In A, $P=0.04$ for PKA d.n., <0.001 for *PKA* RNAi, 0.4 for *amn* RNAi #5606 and 0.052 for *amn* RNAi #25797. In B, $P=0.12$ for PKA d.n., 0.048 for *PKA* RNAi, 0.4 for *amn* RNAi #5606 and 0.18 for *amn* RNAi #25797. (C) Total GFP-Imp and Me31B-GFP levels measured from confocal images of 37-40 day-old MB γ neurons. Data point represents the mean value obtained from three to four independent replicates and error bars s.e.m. Values were normalized to the control condition. At least 12 fields were imaged per replicate and per condition. n.s. stands for not significant (unpaired, two-sided Mann-Whitney test). (D) Normalized numbers of Imp-containing granules (per surface area) in 30 day-old (aged) control brains (left) and brains with reduced PKA activity. PKA dn stands for PKA dominant negative and corresponds to expression of a kinase dead variant. At least 10 fields were imaged per condition. Three replicates were performed and the mean value of each replicate is indicated as a symbol (triangle). Data points were color-coded based on the experimental replicate they belong to. ***, $P<0.001$ (one-way ANOVA test with Sidak's multiple comparison tests). Source data are provided as a Source Data file.

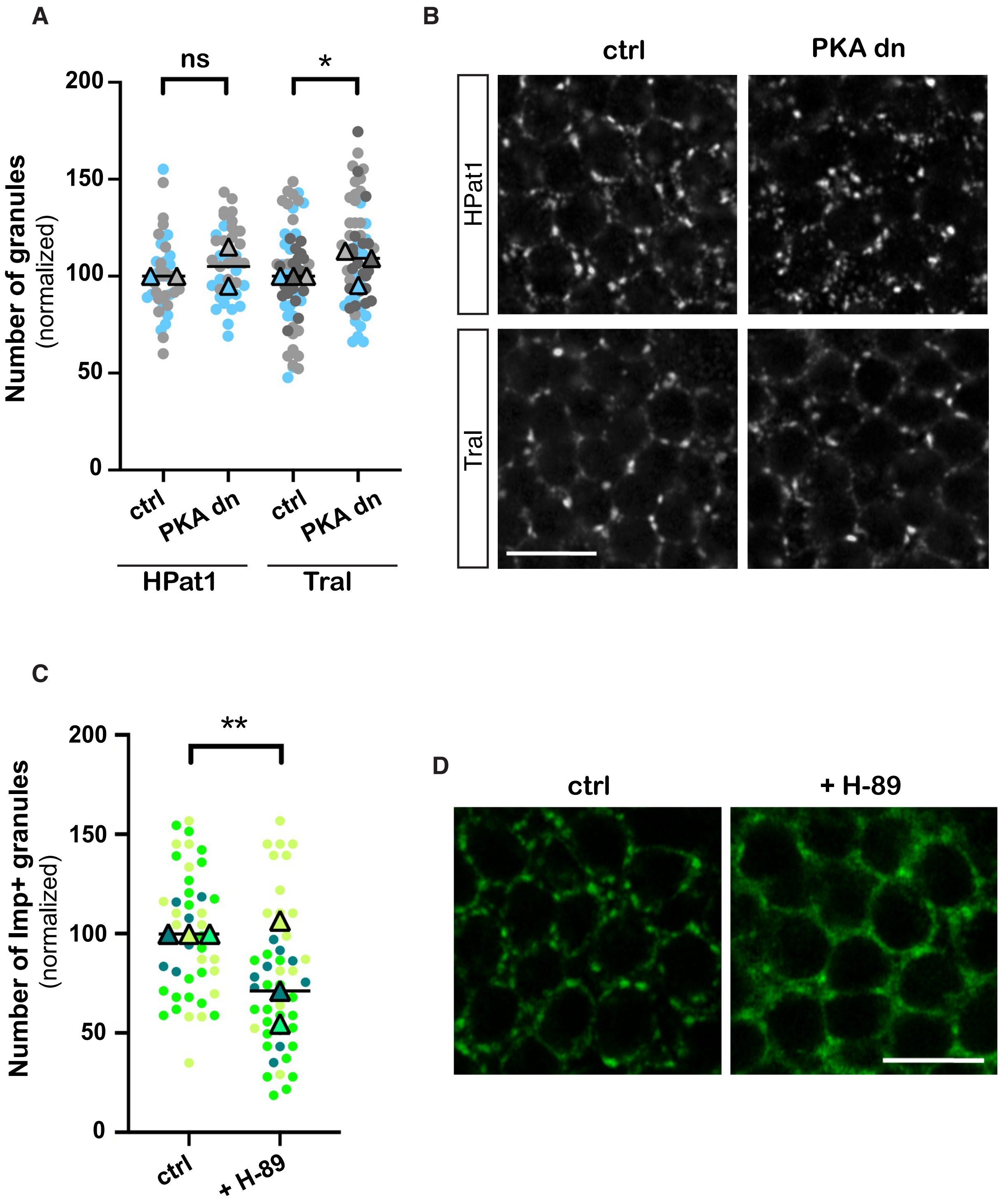
A
B
ns
*
200
150
100
50
0
Number of granules
(normalized)
ctrl
PKA dn
ctrl
PKA dn
HPat1
Tral
ctrl
PKA dn
HPat1
Tral
C
D
**
Number of Imp+ granules
(normalized)
ctrl
+ H-89
ctrl
+ H-89

Supplementary Figure 6. PKA promotes Imp condensation acutely, and specifically.

(A) Number of granules (per surface area) containing HPat1 (left) or Tral (right) upon inhibition of PKA in 30 day-old (aged) brains. Values were normalized to controls. Two to three replicates were performed and the mean value of each replicate is indicated as a symbol (triangle). Data points were color-coded based on the experimental replicate they belong to. At least 20 fields were imaged per condition. *, $P<0.05$ (unpaired, two-sided t-test). n.s. stands for not significant. $P= 0.11$ for HPat1 and 0.02 for Tral. (B) Cell bodies of 30 day-old MB γ neurons expressing (right) or not (left) a PKA kinase dead variant using tub-Gal80ts; OK107-Gal4. Samples were stained with anti-HPat1 (upper panel) or anti-Tral (lower panel) antibodies. Scale bar: 5 μm. (C) Numbers of Imp-containing granules (per surface area) in control (left) and H-89-treated (right) brains of 35-38 day-old flies. Values were normalized to the control condition. Three replicates were performed and the mean value of each replicate is indicated as a symbol (triangle). Data points were color-coded based on the experimental replicate they belong to. At least 6 fields were imaged per condition. **, $P<0.01$ (unpaired, two-sided t-test). $P=0.002$. (D) Cell bodies of 37-38 day-old MB γ neurons expressing endogenous GFP-Imp (green) and treated (right), or not (left), with the PKA inhibitor H-89. Scale bar: 5 μm. Source data are provided as a Source Data file.

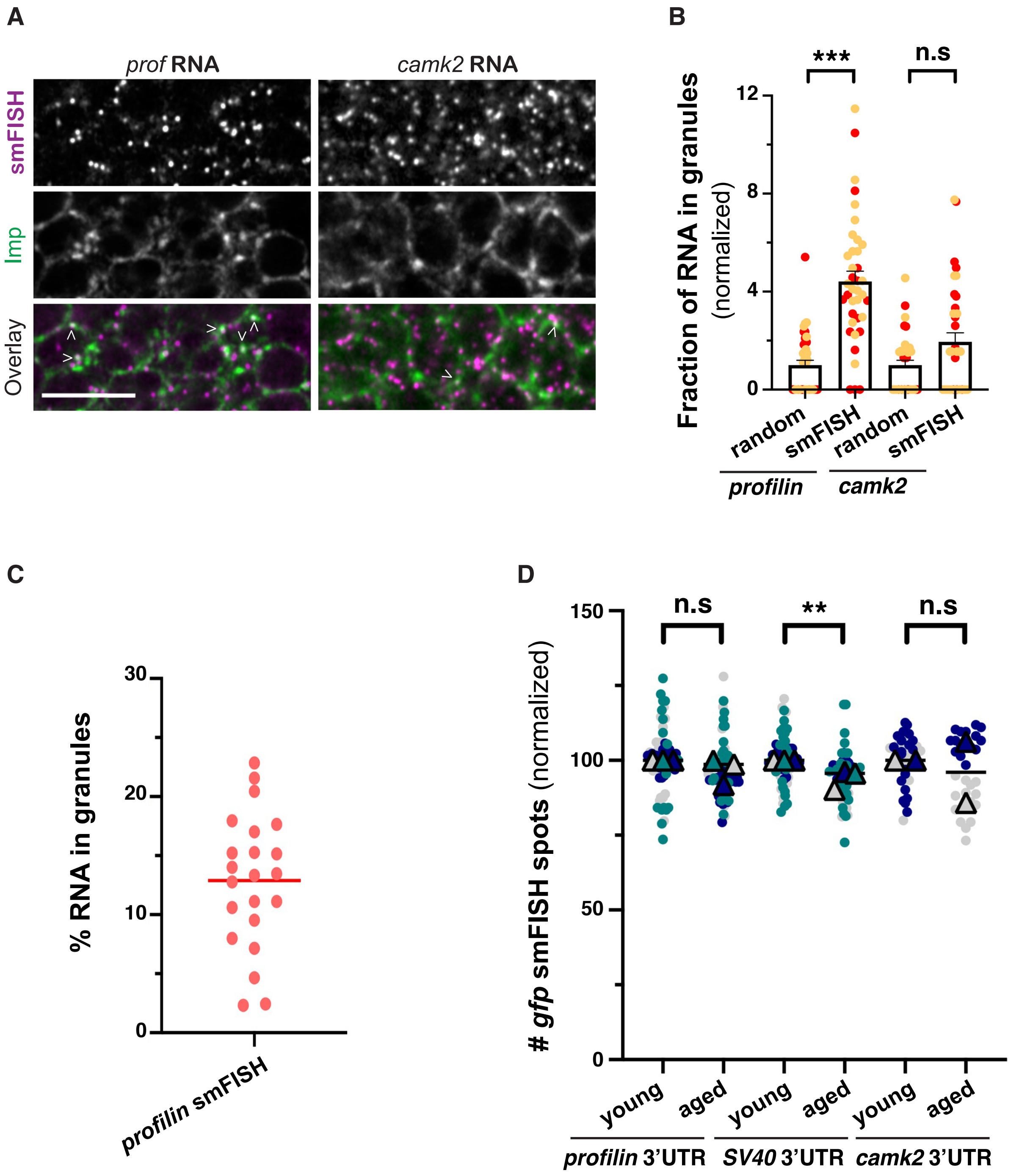
A
prof RNA
camk2 RNA
smFISH
Imp
Overlay
B
***
n.s
Fraction of RNA in granules
(normalized)
12
8
4
0
random
smFISH
random
smFISH
profilin
camk2
C
% RNA in granules
30
20
10
0
profilin smFISH
D
n.s
**
n.s
gfp smFISH spots (normalized)
150
100
50
0
young
aged
young
aged
young
aged
profilin 3'UTR
SV40 3'UTR
camk2 3'UTR

Supplementary Figure 7. Association of *profilin* RNA with granules and levels of *gfp-profilin*-3'UTR transcripts.

(A) Cell bodies of GFP-Imp-expressing MB γ neurons from aged brains. *profilin* (left) *and camk2* (right) smFISH signals are shown in white (upper panel) and magenta (overlay; lower panel). GFP-Imp signals are shown in white in the middle panel and green in the overlay. Arrowheads point to *profilin* RNA co-localizing with granules. Scale bar: 5 μm. (B) Relative fractions of RNA molecules found in granules. The fraction of smFISH spots co-localizing with Imp+ granules was estimated for each imaged field using the JACoP plugin of Fiji (see Method). Values were normalized to their respective random control, in which non-specific levels of colocalization were assessed after rotating smFISH images by 90°. Two replicates were performed. Data points were color-coded based on the experimental replicate they belong to. Main bars correspond to mean values and error bars to s.e.m. ***, $P<0.001$ (Kruskall-Wallis test with Dunn's post-test). n.s. stands for not-significant (P=0.2). n=38 for *profilin* mRNA and 36 for *camk2* mRNA. (C) Manually estimated percentages of *profilin* smFISH spots co-localizing with Me31B-GFP granules in aged brains. Percentages were estimated on individual imaged fields (n=23). (D) Number of *gfp* smFISH spots (per surface area) in 1-2 day- (young) and 37-40 day- (aged) old brains expressing EGFP-*profilin* (left), *SV40* (middle) or *camk2* (right) 3'UTR reporters. Values were normalized to the young condition for each reporter. Three replicates were performed and the mean value of each is indicated as a triangle. Data points were color-coded based on the replicate they belong to. At least 12 fields were imaged per condition. **, $P<0.01$ (one-way ANOVA test with Sidak's multiple comparison tests). n.s. stands for not significant. P=0.07 for *profilin* reporter, 0.007 for *SV40* and 0.36 for *camk2*. Source data are provided as a Source Data file.

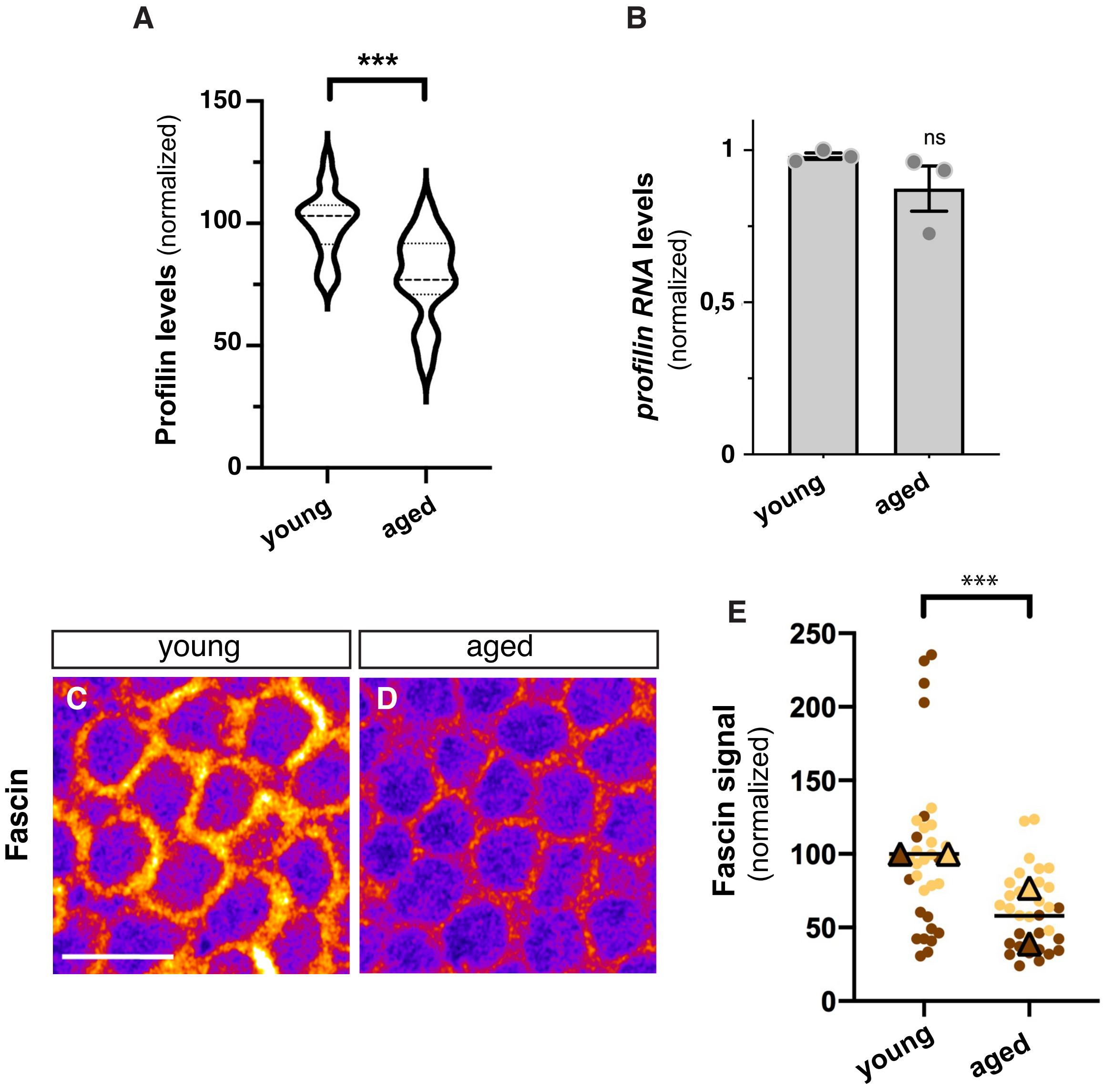
A
B
***
150
100
50
0
Profilin levels (normalized)
young
aged
1
0,5
0
ns
profilin RNA levels
(normalized)
young
aged
E
young
aged
C
D
Fascin
***
250
200
150
100
50
0
Fascin signal
(normalized)
young
aged

Supplementary Figure 8. Expression of endogenous *profilin* and organization of the F-actin cytoskeleton upon aging.

(A) Normalized Profilin protein levels measured after immunostaining on 1-2 day (young) or 37-38 (aged) day-old brains. ***, $P<0.001$ (unpaired, two-sided Mann Whitney test). (B) Endogenous *profilin* RNA levels. RNA levels were quantified by quantitative RT-PCR and normalized to those of *rp49* and *rpl7*. n.s. stands for not significant (unpaired, two-sided Mann-Whitney test; n=3; $P=0.4$). Error bars correspond to s.e.m (biological replicates). (C,D) Cell bodies of 1-2 day- (young) and 35-38 day- (aged) old MB γ neurons expressing a mcherry-Fascin fusion protein under the control of the OK107-Gal4 driver. (E) Distribution of Fascin signal intensities. Values were normalized to the young condition. Two replicates were performed and the mean value of each replicate is indicated as a symbol (triangle). Data points were color-coded based on the experimental replicate they belong to. At least 14 fields were imaged per condition. ***, $P< 0.001$ (unpaired, two-sided t-test on individual data points). Complete genotype: UAS-RFP-Fascin/+;OK107-Gal4/+. Source data are provided as a Source Data file.

A

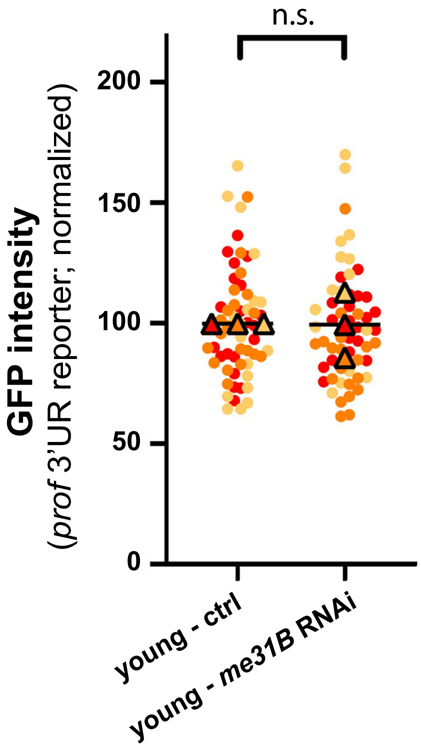
n.s.
200
150
100
50
0
GFP intensity
(prof 3'UR reporter; normalized)
young - ctrl
young - me31B RNAi

B

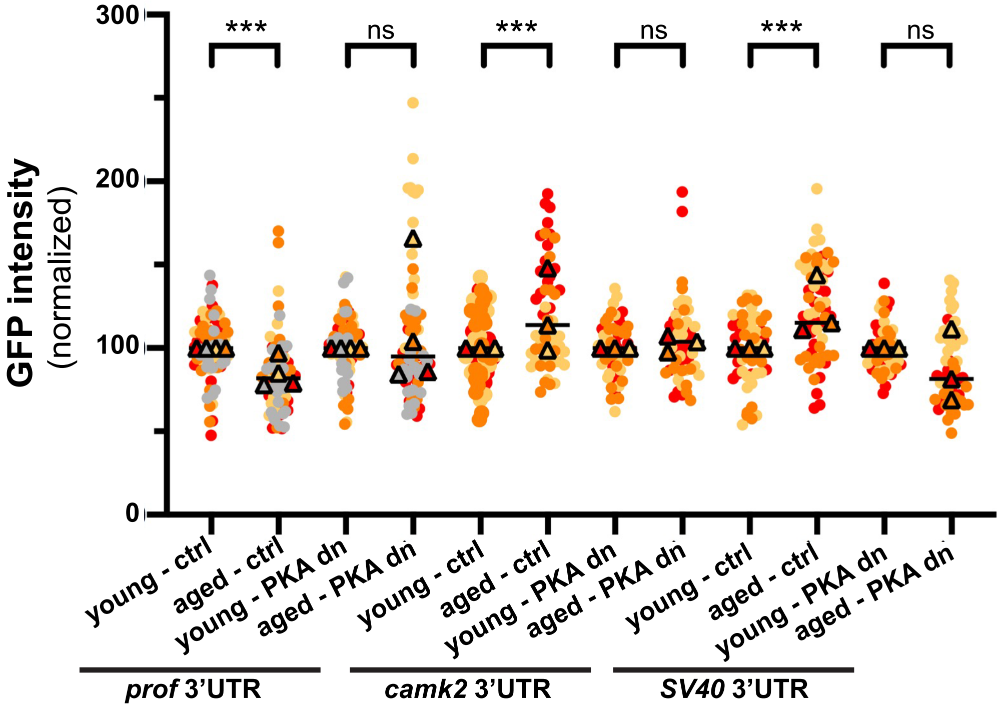
300
200
100
0
***
ns
***
ns
***
ns
GFP intensity
(normalized)
young - ctrl
aged - ctrl
young - PKA dn
aged - PKA dn
young - ctrl
aged - ctrl
young - PKA dn
aged - PKA dn
young - ctrl
aged - ctrl
young - PKA dn
aged - PKA dn
prof 3'UTR
camk2 3'UTR
SV40 3'UTR

Supplementary Figure 9. Impact of *me31B* RNAi and PKA inhibition on RNA translation.

(A) GFP signal intensities measured from 1-2 day-old brains expressing EGFP-*profilin* 3'UTR together with a *me31B* RNAi construct (right). n.s. stands for not significant (unpaired, two-sided t-test). (B) GFP signal intensities measured from 1-2 day- (young) and 37-38 day- (aged) old brains expressing EGFP-*profilin* 3'UTR (left), EGFP-*camk2* 3'UTR (middle) or EGFP-*SV40* 3'UTR (right) together with a PKA dominant negative construct (PKA dn). Note that in these experiments, reporter and PKA dn constructs were expressed constitutively using the OK107-Gal4. Values were normalized to the young conditions. In A, B, three to four replicates were performed and the mean value of each replicate is indicated as a symbol (triangle). Data points were color-coded based on the experimental replicate they belong to. At least 12 fields were imaged per condition. ***, $P < 0.001$ (one-way ANOVA test with Sidak's multiple comparison tests). n.s. stands for not significant. P=0.33 for *profilin* and PKA d.n., 0.96 for *camk2* and PKA d.n., 0.09 for *SV40* and PKA d.n. and <0.001 for all other comparisons. Source data are provided as a Source Data file.

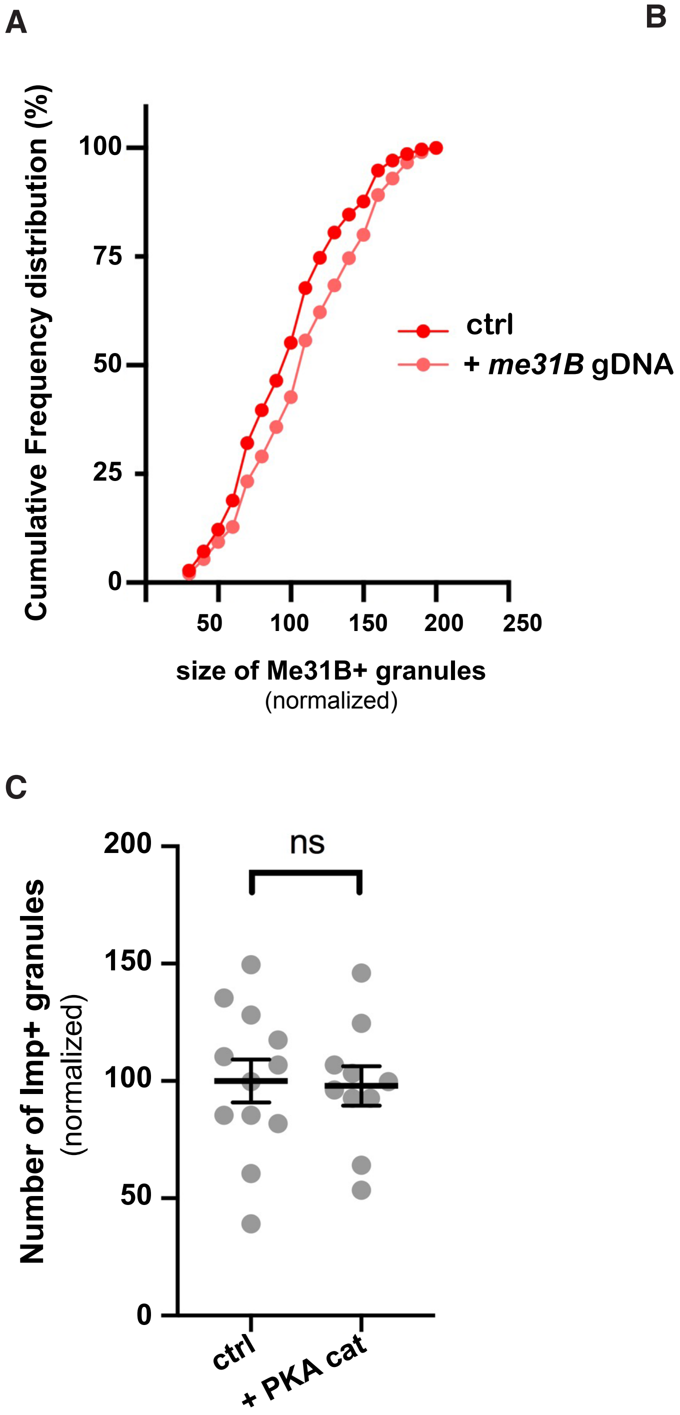
A
Cumulative Frequency distribution (%)
100
75
50
25
0
50
100
150
200
250
size of Me31B+ granules
(normalized)
ctrl
+ me31B gDNA
B
PKA activity (normalized)
300
200
100
0
***
young
aged
C
Number of Imp+ granules
(normalized)
200
150
100
50
0
ns
ctrl
+ PKA cat

Supplementary Figure 10. Modulation of Me31B levels and of PKA activity in young flies

(A) Sizes of Me31B-containing granules in MB γ neurons of 1-2 day- (young) containing an additional copy of *me31B* (+ *me31B* gDNA). Values were normalized to the control condition. No significant difference could be observed when comparing to control (ctrl) flies (unpaired t-test). (B) PKA activity in MB γ neurons of 1-2 day- (young) and 35-38 day- (aged) flies. PKA activity was measured using the GFP-PKA-SPARK reporter, by calculating the fraction of GFP proteins found in condensates in the cell bodies of MB neurons. Values were normalized to the young condition. Three replicates were performed and the mean value of each replicate is indicated as a symbol (triangle). Data points were color-coded based on the experimental replicate they belong to. At least 16 fields were imaged per condition. One outlier data point was omitted from the graph (but was considered to calculate the mean of the corresponding replicate and to perform statistical tests). ***, $P<0.001$ (unpaired, two-sided t-test). Complete genotype: UAS-GFP-PKA-SPARK/+;OK107-Gal4/+. (C) Number of Imp-containing granules (per surface area, normalized) in 1-2 day-old flies over-expressing the catalytic subunit of PKA (PKA-C1, indicated as PKA cat). Means and s.e.m are indicated as black lines. No significant difference could be observed when comparing to control (ctrl) flies (unpaired, two-sided t-test; n=12 for controls and 10 for PKA cat; $P=0.87$). Complete genotype: UAS-PKA-C1/+; OK107-Gal4/+. One replicate was performed. Source data are provided as a Source Data file.

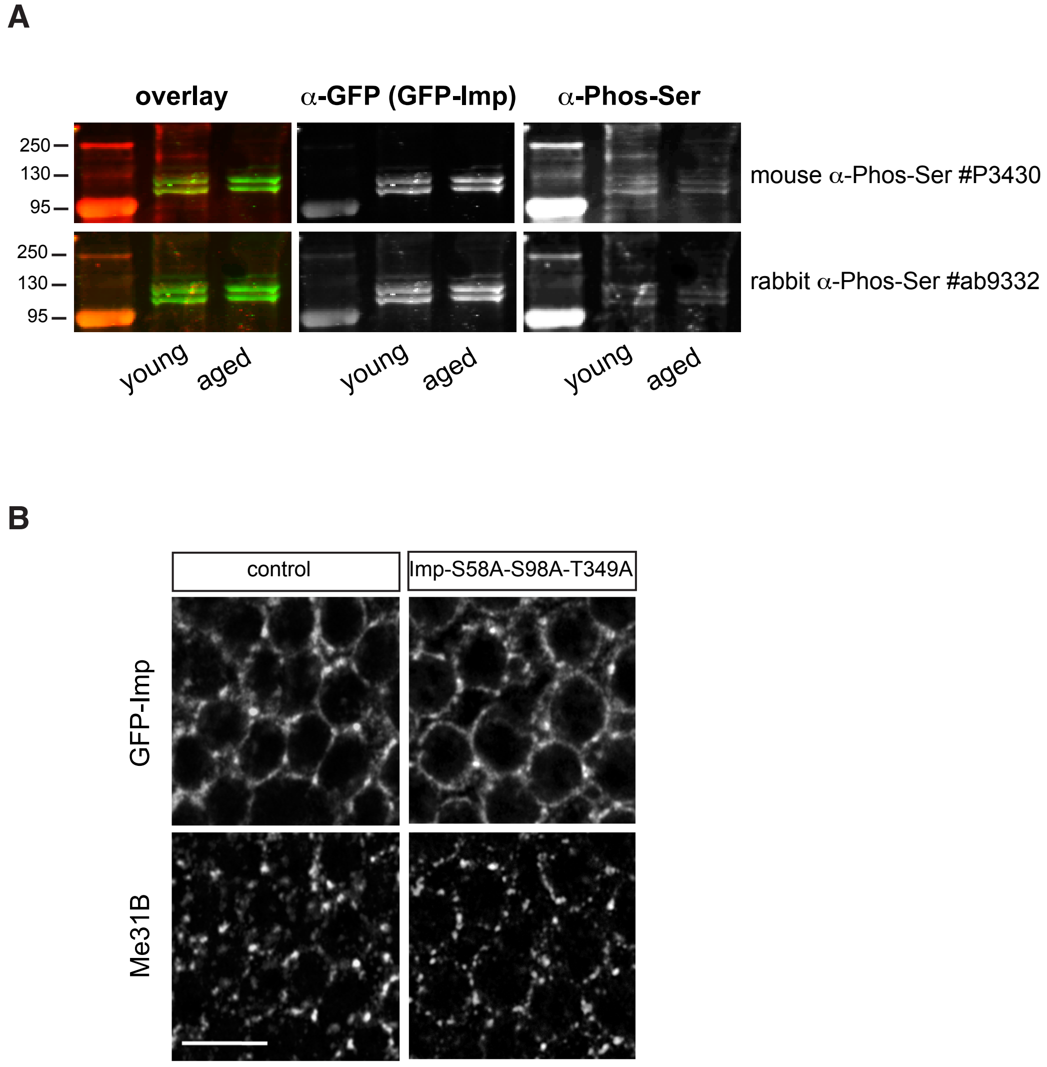
A
overlay
α-GFP (GFP-Imp)
α-Phos-Ser
250
130
95
mouse α-Phos-Ser #P3430
rabbit α-Phos-Ser #ab9332
young
aged
B
control
Imp-S58A-S98A-T349A
GFP-Imp
Me31B

Supplementary Figure 11. Imp is phosphorylated on Serines, but the S58A, S98A and T349A putative PKA phosphorylation sites are dispensable for Imp condensation.

(A) Western-Blot performed on fractions recovered after immuno-precipitation of GFP-Imp proteins from head lysates. Lysates were prepared from 1-2 day-old (young) or 37-40 day- (aged) old flies. Two different anti-phos-Ser antibodies were used for the Western-Blot: a mouse anti-Phos-Ser (Sigma #P3430; upper panel) and a rabbit anti-Phos-Ser (Abcam #ab9332; lower panel). Both revealed bands co-localizing with the GFP-Imp ones. Two independent replicates were performed. (B) Cell bodies of MB γ neurons from 37-40 day-old control GFP-Imp (left) and mutant GFP-Imp-S58A-S98A-T349A (right) brains stained with anti-Me31B antibodies. Imp condensed to a similar degree in both conditions. Two independent replicates were performed. Scale bar: 5 μm. Source data are provided as a Source Data file.

Supplementary Data 1. List of lines tested in the selective screen for modifyers of Imp condensation					
**Pathway**	**Gene**	**Construct**	**Stock reference**	**Source**	**Modification of Imp condensation in aged flies ?**
**ROS**	Sod1	UAS-Sod1.RNAi	24491	BDSC	no
		UAS-Sod1	33605	BDSC	no
		UAS-Sod1 RNAi	36804	BDSC	no
		UAS-Sod2 RNAi	25969	BDSC	no
	Sod and catalase	UAS cat/cyo; UAS sod/tm		Bolan Laura	no
	Catalase	UAS-Cat.A	24621		no
**chaperones**	hsp 22	UAS hsp22 RNAi (GD)	v43632	VDRC	no
		UAS hsp22	20055	BDSC	no
	Hsc70	UAS_Hsc70wt	5846	BDSC	no
	Atg1	yw,hsflp;UASatg1		Neufeld Thomas	no
**Insulin pathway**	InR	UAS InR DN	8251	BDSC	no
		UAS InR CA	8263	BDSC	no
**Aging-related genes**	sirt2	UAS sirt2 RNAi	31613	BDSC	no
	sirt1	UAS sirt1 RNAi	53697	BDSC	no
		UAS sirt1 (low expression)	44217	BDSC	no
		UAS sirt1 (high expression)	44216	BDSC	no
	Methusellah	UAS mth	64194	BDSC	no
		mth mutant	27896	BDSC	no
		mth RNAi	27495	BDSC	no
		mth RNAi	67829	BDSC	no
	Methyl transferase	UAS Mettl3 RNAi	41590	BDSC	no
**Mitochondrial function**	surf1	UAS surf1 RNAi	51783	BDSC	no
		UAS surf1 RNAi	51783	BDSC	no
	TFAM	UAS TFAM RNAi	26744	BDSC	no
		UAS TFAM RNAi	57742	BDSC	no
	dj1-β	UAS dj1-β	33604	BDSC	no

Supplementary Data 2. Sequences of the probe sets used to detect *profilin*, *egfp* and *camk2* mRNAs.

From 5’ to 3’:

profilin: ccgcaacaccgacgattt, cacacgaaattggcaggg, tcgcactttcgtttcggg, ttgctttaccgcacggcg, gatctggatatg gatcgc, gggtgcggattaagttga, catggtgctttgtttgtc, gtccacataatcttgcca, ctgcgaggccaggagttg, gatgcacgccttggt cac, ccaaatgttgccgtcgtg, tcacctcaaagccactgg, gtttggagagctcctctt, ctggtcaaagccgctgat, gttgctggtgagaccgtc , aaatgtaccgctggccgg, gcggtctgtgccggaaag, ttcatgcagtgcactccg, acgatcacggcttgtgtt, cgggatcctcgtagatgg, tctctaccacggaagcgg, ctattctcctagtacccg, tcatttacggttcgctct, tggtttttcttttcccat, gcaaattctttcttggcc, tcctctgct acacacaaa, gcatttttactcgatcca

***gfp*:** tcctcgcccttgctcaccat, atgggcaccaccccggtgaa, gtcgccgtccagctcgacca, cgctgaacttgtggccgttt, tcgccctcgccctcgccgga, ggtcagcttgccgtaggtgg, cggtggtgcagatgaacttc, ggccagggcacgggcagctt, taggtcagggtggtcacgag, tagcggctgaagcactgcac, gtgctgcttcatgtggtcgg, gcatggcggacttgaagaag, cgctcctggacgtagccttc, gtcgtccttgaagaagatgg, tcggcgcgggtcttgtagtt, ggtgtcgccctcgaacttca, ttcagctcgatgcggttcac, gtcctccttgaagtcgatgc, agcttgtgccccaggatgtt, gtggctgttgtagttgtact, ttgtcggccatgatatagac, caccttgatgccgttcttct, atgttgtggcggatcttgaa, gagctgcacgctgccgtcct, tgttctgctggtagtggtcg, agcacggggccgtcgccgat, caggtagtggttgtcgggca, ttgctcagggcggactgggt, atcgcgcttctcgttggggt, cgaactccagcaggaccatg, agagtgatcccggcggcggt, cttgtacagctcgtccatgc

***camk2*:** gaaaaacgcgtacaggctgc, ccaactcttctttgatgtcg, tcacaattgagaatgctcct, gcagcaaattcaaagccagt, gtctctggcagttaattttt, cttcacgttccagtttttga, tgtaactttctgcagatcct, tgtatactgtcatgtagccg, aaggtagtgatagttctcct, aatgtgatgcatcagcttct, cattttggtggcagtgattg, attctctggtttcagatctc, ccttagccttactagctaat, agaccaaagtcagcgagttt, ctgatgatcgccttgaactt, gagacagatatcctggtgtg, tagggctcctttttcaatac, aagaataactccacatgccc, atcttcatcccaaaagggtg, tttatctgcgagtacagtcg, acggataatcataagctccc, tttagcttctggagtaaccg, gatgttttaaagcctcagct, cacgttcgcgttgacaaatc, ttggcgatgcacaacggaag, tcttgagacagtctacggtt, cgccaacattgtcgtaagta, gttatcatacttctgctcga, gttgattctttgacctgtga, cgtcttcaagagtagtgcta, ggttcctttttatcttctt, acgactgtagtactgcgatc, cttatatcttcaggctcttt, agtttttgcaggacacagta, cactgcgagtttcctatatt, tttcttgtcgtctagctgaa, tgcttcaatcaactgctcag, catcaaagtcgccactgtta, caaaggcagttagatgcgga, attccttctacaaggttacc, gctttgcagtttttaccaag, cttcaccaagtaagtgcaca, gtctcacataggcaatgcaa, tgcttatcaatgtactgcgt, gactgatgggtatgtgcatg, cattctgccatttgttatcg,

attttggcagatgcacttcg, aatcgaatgtagttgctccg.
