# Supplementary material for: RNP components condense into repressive RNP granules in the aging brain
Source: Nat Commun. 2022 May 19;13:2782. doi: 10.1038/s41467-022-30066-4 (PMC9120078; doi:10.1038/s41467-022-30066-4)
Supplement: Supplementary file 3 — Description of Additional Supplementary Files [file 41467_2022_30066_MOESM3_ESM.pdf]

Description of Additional Supplementary Files

File Name: Supplementary Movie 1
Description: **Imp and Me31B coalesce into dynamic multiphase condensates.** Image sequence recorded from 5 day-old GFPImp; Me31B-mTomato/+ intact brains. GFP-Imp is shown in green, Me31B-mTomato in red. Time interval: 10 sec. Scale bar: 0.5 μm.
