# Supplementary material for: RNP components condense into repressive RNP granules in the aging brain
Source: Nat Commun. 2022 May 19;13:2782. doi: 10.1038/s41467-022-30066-4 (PMC9120078; doi:10.1038/s41467-022-30066-4)
Supplement: Supplementary file 5 — Reporting Summary [file 41467_2022_30066_MOESM5_ESM.pdf]

nature portfolio

Corresponding author(s): Florence BESSE

Last updated by author(s): 2022/03/11

Reporting Summary

Nature Portfolio wishes to improve the reproducibility of the work that we publish. This form provides structure for consistency and transparency in reporting. For further information on Nature Portfolio policies, see our Editorial Policies and the Editorial Policy Checklist.

Statistics

For all statistical analyses, confirm that the following items are present in the figure legend, table legend, main text, or Methods section.

n/a	Confirmed	
☐	☒	The exact sample size (*n*) for each experimental group/condition, given as a discrete number and unit of measurement
☐	☒	A statement on whether measurements were taken from distinct samples or whether the same sample was measured repeatedly
☐	☒	The statistical test(s) used AND whether they are one- or two-sided<br>*Only common tests should be described solely by name; describe more complex techniques in the Methods section.*
☐	☒	A description of all covariates tested
☐	☒	A description of any assumptions or corrections, such as tests of normality and adjustment for multiple comparisons
☐	☒	A full description of the statistical parameters including central tendency (e.g. means) or other basic estimates (e.g. regression coefficient) AND variation (e.g. standard deviation) or associated estimates of uncertainty (e.g. confidence intervals)
☐	☒	For null hypothesis testing, the test statistic (e.g. *F*, *t*, *r*) with confidence intervals, effect sizes, degrees of freedom and *P* value noted<br>*Give P values as exact values whenever suitable.*
☒	☐	For Bayesian analysis, information on the choice of priors and Markov chain Monte Carlo settings
☒	☐	For hierarchical and complex designs, identification of the appropriate level for tests and full reporting of outcomes
☒	☐	Estimates of effect sizes (e.g. Cohen's *d*, Pearson's *r*), indicating how they were calculated

Our web collection on statistics for biologists contains articles on many of the points above.

Software and code

Policy information about availability of computer code

Data collection	Zen 2.3 and Metamorph 7.10.1.161 were used to acquire images on laser scanning and spinning disc microscopes respectively.
Data analysis	SPaDe algorithm (spyder3-Phyton 3.6) was used to detect granule property quantification (https://raweb.inria.fr/rapportsactivite/RA2016/morpheme/uid13.html) (De Graeve et al. 2019) and extract quantitative parameters.<br>Image analysis was performed with Fiji/Image J 2.0. Statistical analyses were performed using GraphPad Prism 8.

For manuscripts utilizing custom algorithms or software that are central to the research but not yet described in published literature, software must be made available to editors and reviewers. We strongly encourage code deposition in a community repository (e.g. GitHub). See the Nature Portfolio guidelines for submitting code & software for further information.

Data

Policy information about availability of data

All manuscripts must include a data availability statement. This statement should provide the following information, where applicable:

- Accession codes, unique identifiers, or web links for publicly available datasets
- A description of any restrictions on data availability
- For clinical datasets or third party data, please ensure that the statement adheres to our policy

All data are included in this article as main or supplementary information files and are provided in the Source Data file. Additional information can be obtained from the corresponding author. Source data are provided as a Source Data file.

Field-specific reporting

Please select the one below that is the best fit for your research. If you are not sure, read the appropriate sections before making your selection.

☒ Life sciences ☐ Behavioural & social sciences ☐ Ecological, evolutionary & environmental sciences

For a reference copy of the document with all sections, see nature.com/documents/nr-reporting-summary-flat.pdf

Life sciences study design

All studies must disclose on these points even when the disclosure is negative.

Sample size	No statistical method was used to predetermine sample size. The sample size was based on current standards in the field and previous experimental experience. The information about number of replicates and sample sizes is provided in figure legends and Source Data.
Data exclusions	No data were excluded from the analysis.
Replication	Unless specified in the Figure legend, three independent replicates were performed for each experiments. The superplots representations we have chosen provide an estimation of the variability observed from one replicate to the other. Experiments shown in Figure 8a and 8b were sensitive to temperature and temperature switches and had to be optimized to produce efficient age-dependent translation repression while sufficiently inactivating me31B and PKA.
Randomization	Flies were included into groups based on their genotypes. Cells were included into groups based on the identity of their transfected constructs.
Blinding	Samples were not blinded as quantifications relied on computational softwares (eg:SPADE) or measure of signal intensities, avoiding any subjective assessments of genotype.

Reporting for specific materials, systems and methods

We require information from authors about some types of materials, experimental systems and methods used in many studies. Here, indicate whether each material, system or method listed is relevant to your study. If you are not sure if a list item applies to your research, read the appropriate section before selecting a response.

Materials & experimental systems

n/a	Involved in the study	
☐	☒	Antibodies
☒	☐	Eukaryotic cell lines
☒	☐	Palaeontology and archaeology
☐	☒	Animals and other organisms
☒	☐	Human research participants
☒	☐	Clinical data
☒	☐	Dual use research of concern

Methods

n/a	Involved in the study	
☒	☐	ChIP-seq
☒	☐	Flow cytometry
☒	☐	MRI-based neuroimaging

Antibodies

Antibodies used

- The following antibodies were used for Western-Blots: rabbit anti-GFP (1:2,500; Torrey Pines, #TP401); rabbit anti-phospho serine (3ug/mL; Abcam #ab9332) and mouse anti-phospho serine (1:200; Sigma #P3430).

- The following antibodies were used for immuno-fluorescence: : rabbit α-tmp (1:1000; Medioni et al., 2014); rat α-Imp (1:1,000; Medioni et al., 2014); rabbit α-Me31B (1:3,000; gift from C. Lim); mouse α-Me31B (1:3,000; gift from A. Nakamura); rabbit α- HPat1 (1:1,000; gift from A. Nakamura), rabbit α-Tral (1:1,000; gift from A. Nakamura), rat α-Staufen (1:1,000; gift from A. Ephrussi); rabbit α-GFP (1:1,000; Molecular Probes, A-11122), mouse α-Profilin (1:100; DSHB, chi 1J clone); rabbit α-p62 (1:1,000; gift from Gabor Juhasz), mouse α-Ubiquitin (1:500; Enzo Biosciences Cat # BML-PW8805), rabbit α-PABP (1:1,500; gift from C.Lim), α-rabbit alexa Fluor 488 (1:1000; Thermo Fisher Scientific, # A-21206), α-rabbit alexa Fluor 568 (1:1000; Thermo Fisher Scientific,# A10042), α-rabbit alexa Fluor 647 (1:1000; Thermofisher scientific, # A-31573), α-rat alexa Fluor 488 (1:1000; Thermofisher scientific, # A-21208), α-rat alexa Fluor 568 (1:1000; Thermofisher scientific, # A-11077), α-rat alexa Fluor 647 (1:1000; Thermofisher scientific, # A-21247), α-mouse alexa Fluor 488 (1:1000;Thermofisher scientific, # A-21202), α-mouse alexa Fluor 568 (1:1000; Thermofisher scientific, # A10037), α-rmouse alexa Fluor 647 (1:1000; Thermofisher scientific, #A-31571).

Validation

Descriptions and validations of the Drosophila antibodies can be found on the DHSB website (http://dshb.biology.uiowa.edu/) or in the respective original publications:
Antibodies used for IF:
rat anti-tmp (Medioni et al., 2014; DOI:10.1016/j.cub.2014.02.038) , Original source-Besse lab, iBV,Nice, France.

rabbit anti-tmp (Medioni et al., 2014; DOI:10.1016/j.cub.2014.02.038) ,Original source-Besse lab, iBV,Nice, France.
rat anti-Staufen (Ghosh et al., 2014; DOI:10.1371/journal.pgen.1004455), Original source- Ephrussi lab, EMBL, Heidelberg, Germany.
rabbit anti-Me31B (Lee et al., 2017; DOI:10.1016/j.molcel.2017.03.004), Original source- Nakamura lab, IMEG,Kumamoto, Japan.
-mouse anti-Ubiquitin (Rallis et al,2020;DOI: 10.1016/j.celrep.2020.02.006), Original source- Enzo Biosciences, Cat: # BML-PW88050500, clone name:FK1.
-rabbit anti-p62 (Nezis et al, 2008; DOI: 10.1083/jcb.200711108), Original source- Juhasz lab, ELTE, Budapest, Hungary.
-rabbit anti-GFP (Medioni et al., 2014; DOI:10.1016/j.cub.2014.02.038),Original source- Thermo Fisher Scientific, Catalog # A-11122
-rabbit anti-Tral (Vijayakumar et al, 2019; DOI:10.1038/s41467-019-10554-w), Original source- Nakamura lab, IMEG,Kumamoto, Japan.
-rabbit anti-Hpat1 (Pradhan et al, 2012; DOI:10.1242/jcs.113043), Original source- Nakamura lab, IMEG,Kumamoto, Japan.
-mouse anti-Me31B (Formicola et al, 2021; DOI: 10.75S4/eLife.65742), Original source- Nakamura lab, IMEG,Kumamoto, Japan.
rabbit anti-PABP (Lee et al, 2017; DOI:10.1016/j.molcel.2017.03.004), Original source-Chunghun Lim's Lab, UNIST, Ulsan, South Korea
-mouse anti-profilin (Medioni et al., 2014; DOI:10.1016/j.cub.2014.02.038), Original source- DSHB, chi 1J clone

Antibodies used for WB:
-rabbit anti-phospho serine (Lei et al, 2020; 10.1038/s41389-020-0208-1), Original source-Abcam #ab9332
-mouse anti-phospho serine (Wurm et al, 201S(; DOI:10.1101/gad.249748.114), Original source-Sigma #P3430,CLONE PSR-45.
-rabbit anti-GFP (Vijayakumar et al, 2019; DOI:10.1038/s41467-019-10554-w) Original source- Torrey Pines, #TP401.

Secondary antibodies:
-anti-mouse alexa Fluor 488- Original source-Thermofisher scientific, Catalog # A-21202
-anti-rabbit alexa Fluor 488- Original source-Thermofisher scientific, Catalog # A-21206
-anti-rat alexa Fluor 488- Original source-Thermofisher scientific, Catalog # A-21208
-anti-mouse alexa Fluor 568- Original source-Thermofisher scientific, Catalog # A10037
-anti-rabbit alexa Fluor 568- Original source-Thermofisher scientific, Catalog # A10042
-anti-rat alexa Fluor 568- Original source-Thermofisher scientific, Catalog # A-11077
-anti-mouse alexa Fluor 647- Original source-Thermofisher scientific, Catalog #A-31571
-anti-rabbit alexa Fluor 647- Original source-Thermofisher scientific, Catalog # A-31573
-anti-rat alexa Fluor 647- Original source-Thermofisher scientific, Catalog # A-21247

Animals and other organisms

Policy information about studies involving animals; ARRIVE guidelines recommended for reporting animal research

Laboratory animals	w1118-derived Drosophila melanogaster individuals were used in our study. Unless specified, males and females were indistinguishly used. Individuals of different ages were used (2-day old and 35-38 day-old), and the age is specified for each experiment in the Figure legend.
Wild animals	No wild animals were used in the study
Field-collected samples	No field-collected samples were used in the study.
Ethics oversight	This study does not require any ethical approval

Note that full information on the approval of the study protocol must also be provided in the manuscript.
